# Reduced Long-Term Relative Survival in Females and Younger Adults Undergoing Cardiac Surgery: A Prospective Cohort Study

**DOI:** 10.1371/journal.pone.0163754

**Published:** 2016-09-28

**Authors:** Tone Bull Enger, Hilde Pleym, Roar Stenseth, Guri Greiff, Alexander Wahba, Vibeke Videm

**Affiliations:** 1 Department of Laboratory Medicine, Children’s and Women’s Health, Faculty of Medicine, NTNU-Norwegian University of Science and Technology, Trondheim, Norway; 2 Department of Circulation and Medical Imaging, Faculty of Medicine, NTNU-Norwegian University of Science and Technology, Trondheim, Norway; 3 Clinic of Anaesthesia and Intensive Care, St. Olavs University Hospital, Trondheim, Norway; 4 Department of Cardiothoracic Anaesthesia and Intensive Care, St. Olavs University Hospital, Trondheim, Norway; 5 Clinic of Cardiothoracic Surgery, St. Olavs University Hospital, Trondheim, Norway; 6 Department of Immunology and Transfusion Medicine, St. Olavs University Hospital, Trondheim, Norway; University of Milano, ITALY

## Abstract

**Objectives:**

To assess long-term survival and mortality in adult cardiac surgery patients.

**Methods:**

8,564 consecutive patients undergoing cardiac surgery in Trondheim, Norway from 2000 until censoring 31.12.2014 were prospectively followed. Observed long-term mortality following surgery was compared to the expected mortality in the Norwegian population, matched on gender, age and calendar year. This enabled assessment of relative survival (observed/expected survival rates) and relative mortality (observed/expected deaths). Long-term mortality was compared across gender, age and surgical procedure. Predictors of reduced survival were assessed with multivariate analyses of observed and relative mortality.

**Results:**

During follow-up (median 6.4 years), 2,044 patients (23.9%) died. The observed 30-day, 1-, 3- and 5-year mortality rates were 2.2%, 4.4%, 8.2% and 13.8%, respectively, and remained constant throughout the study period. Comparing observed mortality to that expected in a matched sample from the general population, patients undergoing cardiac surgery showed excellent survival throughout the first seven years of follow-up (relative survival ≥ 1). Subsequently, survival decreased, which was more pronounced in females and patients undergoing other procedures than isolated coronary artery bypass grafting (CABG). Relative mortality was higher in younger age groups, females and patients undergoing aortic valve replacement (AVR). The female survival advantage in the general population was obliterated (relative mortality ratio (RMR) 1.35 (1.19–1.54), p<0.001). Increasing observed long-term mortality seen with ageing was due to population risk, and younger age was independently associated with increased relative mortality (RMR per 5 years 0.81 (0.79–0.84), p<0.001)).

**Conclusions:**

Cardiac surgery patients showed comparable survival to that expected in the general Norwegian population, underlining the benefits of cardiac surgery in appropriately selected patients. The beneficial effect lasted shorter in younger patients, females and patients undergoing AVR or other procedures than isolated CABG. Thus, the study identified three groups that need increased attention for further improvement of outcomes.

## Introduction

Factors associated with mortality following adult cardiac surgery can be patient- or procedure-related [1]. During the last decades, there has been a consistent focus on improving surgical techniques, pre- and postoperative care, resulting in reduced operative mortality [2, 3]. However, parallel to therapeutic advances, life expectancy in industrialized countries increases; people tend to get older and have increased comorbidity and more health issues when being referred to cardiac surgery [4]. Thus, it is desirable to obtain information on late mortality in order to capture whether there is a sustained mortality reduction following cardiac surgery.

Recent studies have provided reports of all-cause mortality and conveyed information on potential predictors of long-term mortality following cardiac surgery [2, 5–12]. Age has consistently emerged as the most important risk factor. However, long-term mortality in cardiac surgery patients must be seen in context with the background mortality in the general population. Reports from the 1980–90s adopted relative survival analysis in order to assess the excess and relative mortality associated with cardiac disease in operated patients [13–18]. Since then, techniques and equipment have evolved, and the introduction of endovascular- and catheter-based methods together with changing patient demographics might have influenced the target population for cardiac surgery.

Even though cardiac surgery has shown improved short-term outcomes over the last decades, it is seldom curative. Patients undergoing cardiac surgery suffer from severe cardiac disease and usually have several cardiovascular risk factors and co-existing comorbidities. We therefore hypothesized that long-term survival following cardiac surgery has remained unchanged. The aim of this study was to analyse observed and relative long-term survival in patients who underwent cardiac surgery in Trondheim, Norway, from 2000 through 2014. We have explored potential prognostic factors for long-term mortality for a follow-up period of up to 14 years, with special focus on the effects of age, gender and surgical procedure.

## Methods

### Trondheim Heart Surgery Database

Since 1992, adult patients undergoing cardiac surgery in Trondheim have been registered consecutively into the Trondheim Heart Surgery Database as part of the local quality-assurance work. Patient- and procedure-related preoperative characteristics, intraoperative and postoperative events and factors, as well as laboratory values have been registered prospectively. The present study was part of the Cardiac Surgery Outcome Study (CaSOS), which has used the database as a foundation for investigating different complications following adult cardiac surgery. Previously published investigations include risk assessment for prolonged postoperative ventilation [19], increased length of stay in the intensive care unit [20], postoperative heart dysfunction [21, 22], short-term mortality [23], postoperative fluid overload [24], postoperative acute kidney injury [25] and postoperative bleeding complications [26, 27]. CaSOS was approved by the Norwegian Data Inspectorate and the Regional Research Ethics Committee in Medicine (project number 4.2007.1528), Trondheim, Norway on 27 June 2007. The need for informed consent was waived up to April 2008, after which all patients have given their informed consent.

The present part of CaSOS was based on consecutive patients who underwent cardiac surgery in Trondheim, Norway between 1.1.2000 and 31.12.2014. Only the first entry into the data registry during the study period was used for the survival analyses. Patients undergoing off-pump coronary artery bypass (n = 130), transcatheter aortic valve insertion (TAVI, n = 109) and surgery for a thoraco-abdominal aortic aneurysm (n = 22) were excluded from the CaSOS database. As remaining cardiac surgery patients still comprise a heterogeneous group, a subgroup analysis was performed where patients undergoing isolated coronary artery bypass grafting (CABG, n = 5,648), isolated aortic valve replacement (AVR, n = 726) or combined AVR and CABG (n = 829) were compared.

### Endpoint

Data on cause and date of death through December 2014 were obtained through linkage to the Norwegian Cause of Death Registry. Causes of death were provided according to the International Statistical Classification of Diseases and Related Health Problems, 10th Revision (ICD-10) [28]. The unique national registration numbers assigned to each Norwegian citizen enabled accurate linkage. 42 (<0.05%) temporary residents could not be coupled to the Cause of Death Registry, leaving 8,564 patients for the analysis. The primary endpoint of this study was all-cause mortality, referred to as observed long-term mortality. The secondary endpoint was to explore mortality specifically seen in cardiac surgery patients, estimated as relative long-term mortality (see below).

### Statistical analysis

Temporal trends were analysed across year of surgery continuously as well as categorized into 3 time periods (2000–2004, 2005–2009 and 2010–2014). Unless otherwise specified, categorical variables are described as n (%); continuous variables as median (95% confidence intervals (CI)). Differences across time periods were tested using the χ^2^ and Kruskal-Wallis tests for categorical and continuous data, respectively. Changes in mortality rates during the study period were assessed with a chi-square test for departure from the trend line [29]. P-values <0.05 were considered significant. All statistical analyses were performed with Stata (version 13.1, StataCorp LP, Lakeway Drive, USA), Minitab 17 (Minitab Ltd, Coventry, UK) and R (version 3.2.2 64x, R Foundation, http://www.r-project.org).

#### Observed survival and mortality

Observed cumulative survival and hazard rates were calculated using the Kaplan-Meier and Nelson-Aalen estimators, respectively. Univariate survival analyses, with time since operation as the time variable and death (no/yes) as the event, were performed with the Kaplan-Meier method and log-rank test. Different interventions were compared pairwise while correcting for multiple comparisons using the Bonferroni method. As a sensitivity analysis, calculations were repeated for inclusion up to 2006 or 2010, as well as when using cardiovascular death (ICD-10 chapter IX, block I00-I99) as the outcome variable.

#### Relative survival and mortality

Long-term survival and mortality in cardiac surgery patients must be seen in context with that expected in the general population. Relative survival was calculated as the ratio between the observed and expected survival rates [30] and presented graphically over follow-up time. For the complete follow-up time, relative mortality was calculated as the ratio between the observed and expected number of deaths (multiplicative hazard model), providing so-called standardized mortality ratios (SMR). Expected survival and mortality rates were calculated from lifetables compiled from the Norwegian population stratified on age, sex and calendar year, obtained from the Human Mortality Database [31]. Subgroup analyses were performed across gender and pre-defined age groups (<60 years, 60–69 years, 70–79 years and ≥ 80 years).

#### Predictors of observed and excess mortality

Previous studies have pointed out differences in predictors of short- and long-term mortality [6]. Thus, after estimating observed and relative mortality rates, patients who died within 30 days postoperatively, classified as short-term mortality, were excluded from the analysis of prognostic factors.

Potential predictors of observed mortality were investigated using multivariate Cox proportional hazards (PH) modelling. The selection of candidate predictor variables was guided by clinical knowledge and literature, a method recommended to avoid overfitting and confounders as found with selection based on univariate analyses [32]. General demographics (age, gender, body mass index), procedure-related factors (surgical procedure, redo-operation, emergency level), comorbidity and smoking (never/former vs. current smoker) were included into the models block-wise. Surgical procedures were categorized in accordance with EuroScore II’s definition into isolated CABG, 1 non-CABG procedure, 2 surgical procedures or ≥ 3 surgical procedures, where isolated CABG was defined as the reference category [3]. As cardiac surgery patients still constitute a heterogeneous group, a sensitivity analysis was performed by including patients only undergoing CABG and/or AVR.

A secondary analysis was performed to further investigate female gender as a risk factor for long-term mortality. Gender differences in preoperative risk factors were compared with the Mann-Whitney U-test or χ^2^ test for continuous and categorical variables, respectively. Thereafter, a balancing propensity score was developed using logistic regression with gender as the outcome, including the following explanatory variables: Age, body mass index, smoking status, diabetes, hypertension, preoperative history of atrial fibrillation, peripheral vascular disease, chronic pulmonary disease, previous myocardial infarction hypertension, left ventricular hypertrophy, NYHA functional class, diagnosis of chronic heart failure, kidney disease, preoperative serum creatinine, use of beta-antagonists, statins or diuretics before scheduled for surgery, previous cardiac surgery, emergency level of operation, acute preoperative heart failure, and type of surgical procedure. We used 1:1 greedy matching with a calliper width 0.25*the standard deviation of the propensity score to form female-male pairs. Covariate balance was evaluated using standardized differences, where an absolute standardized difference in the covariate mean for a variable ≤ 10% indicated acceptable balance. Analyses were performed using boost [33] and psmatch2 [34] programs in Stata. Following adequate balance of preoperative risk factors, Cox PH modelling for all-cause and cardiovascular mortality was repeated in the matched dataset.

Deviations from the proportionality assumption were assessed graphically and by inclusion of interaction terms between the predictors and time. Separate parameter estimates for pre-specified time periods (<1, 1–5 and >5 years) were compared in order to assess time-dependent effects. Model fit and complexity were compared using log likelihood, the Bayesian and Akaike information criterions (BIC and AIC, respectively). The likelihood ratio test was used to guide final model selection. Goodness-of-fit was evaluated with Harrell’s concordance (C) statistic and Somer’s D correlation coefficient, both measures of the concordance of ranked predicted and observed outcomes [35].

In order to evaluate factors associated with long-term relative mortality, we applied multiplicative modelling of relative mortality as described by Pohar *et al*. [36, 37] using the relsurv package in R [38]. Goodness-of-fit was tested by means of the Brownian Bridge process. Differences in relative mortality between patients with different covariate levels are expressed as relative mortality ratios (RMR).

## Results

### Temporal trend analysis

**Fig 1:**
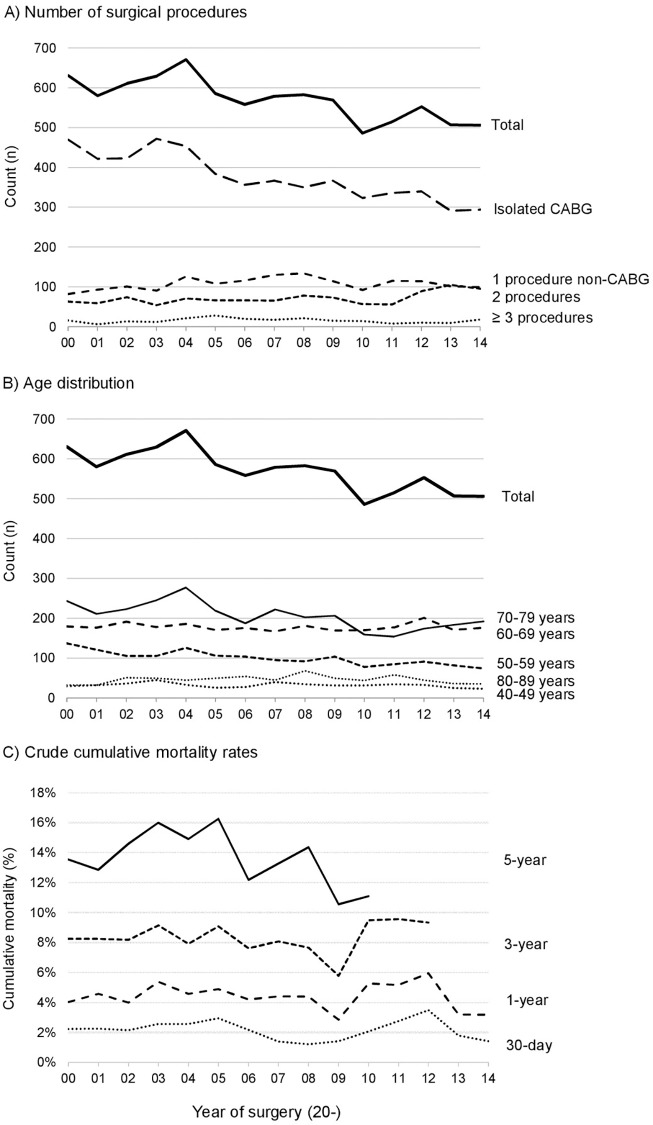
Overview over cardiac operations performed at the Department of Cardiothoracic Surgery, Trondheim, Norway, from 2000 through 2014. A) Stratified on the surgical procedure(s) performed. B) Stratified on age at operation day. For A and B, the total number of procedures is given as reference. C) Cumulative observed mortality rates 30 days, 1, 3 and 5 years following surgery.

Of the 8,564 patients undergoing cardiac surgery in Trondheim from 2000 through 2014, 2,211 (25.8%) were female. The mean yearly number of patients who underwent cardiac surgery was 571 (minimum 486-maximum 671). The annual reduction of total cases was reflected by a steady decline in the number of isolated CABG performed, from 470 (74.5% of yearly procedures) in 2000 to 294 (58.1%) in 2014 (Fig 1A). The reduction in CABG was not compensated by an increase in other procedures.

Median age at the time of surgery was 67.8 years and was constant during follow-up (p = 0.25). The distribution of age across the study period is visualized in Fig 1B. Females were significantly older compared to men (71.7 years versus 66.3 years, p<0.001). Further gender differences are summarized in S1 Table.

A detailed comparison of preoperative, surgical and postoperative factors across the study period is provided in S2 Table. Median age, the proportion of females, smokers and acute surgeries remained constant. Patients admitted to cardiac surgery during more recent years presented with better renal function. However, patients tended to present with more comorbid diseases, such as diabetes (11.9–14.8–15.3% for 2000–2004, 2005–2009 and 2010–2014, respectively, p<0.001) and chronic obstructive lung disease (14.2–13.4–19.2%, p<0.001). More patients presented with acute cardiac insufficiency requiring either ionotropic therapy or intra-aortic balloon pump before surgery (0.6–0.9–1.6%, p = 0.001). There was an increasing proportion of patients scheduled for urgent surgery (within 2 weeks) during more recent years (39.7–41.2–43.3%, p = 0.02).

There was a marked increase in the proportion of patients receiving intraoperative red cell transfusion (13.9–18.8–23.9%, p<0.001), inotropic support (24.3–23.5–30.2%, p<0.001) and vasoconstrictor therapy (67.8–92.8–97.4%, p<0.001) intraoperatively. Median duration of cardiopulmonary bypass increased steadily over the study period (72-79-85 minutes, p<0.001). Nevertheless, the incidence of postoperative complications remained unchanged, and there was a reduction in the duration of postoperative hospital stay (7-6-5 days, p<0.001).

### Mortality following cardiac surgery

The median time to censoring was 6.4 years with a maximum of 14.99 years. A total of 2,044 patients (23.9%) died, corresponding to an observed mortality rate of 22.5% and 28.0% for males and females, respectively (p<0.001). Of the patients who survived the first 30 days postoperatively but died within the follow-up time, 47.0% (men: 45.9%, females: 49.5%), were officially classified as suffering a cardiovascular death, as opposed to 92.4% of the patients who died within 30 days postoperatively (n = 184). The overall mean survival time was 11.6 years (95% CI 11.5–11.7).

#### Observed mortality

**Fig 2:**
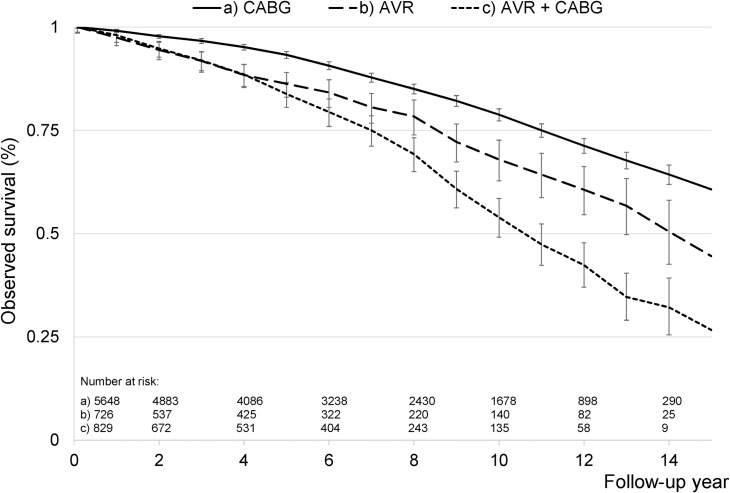
Long-term observed survival. Unadjusted Kaplan-Meier survival curves for patients undergoing coronary artery bypass grafting (CABG) and/or aortic valve replacement (AVR). The number at risk (n) at the start of even follow-up years are provided.

The overall observed 30-day, 1-, 3- and 5-year mortality rates were 2.2%, 4.4%, 8.2% and 13.8%, respectively (Fig 1C). There were no significant changes across follow-up year (p = 0.45, p = 0.78, p = 0.33 and p = 0.88, respectively). Conversely, the observed survival rates calculated by the Kaplan-Meier method were 95.7%, 86.9% and 69.3% after 1, 5 and 10 years, respectively, and differed significantly amongst different surgical interventions as classified by EuroSCORE II (p<0.001, S1 Fig). Similarly, patients undergoing AVR and combined AVR and CABG showed significant differences compared to isolated CABG (Fig 2). Despite a linear increase in observed long-term mortality across patients undergoing isolated CABG, isolated AVR and combined AVR and CABG (HR 1.00, HR 1.39 (1.17–1.64) and 1.59 (1.39–1.82), respectively), observed mortality in AVR-patients undergoing concomitant CABG did not differ significantly from that of isolated AVR (p = 0.48). Comparable trends were seen when using inclusion of patients up to 2006 and 2010, as well as when using cardiovascular death as the outcome variable.

#### Relative survival

**Fig 3:**
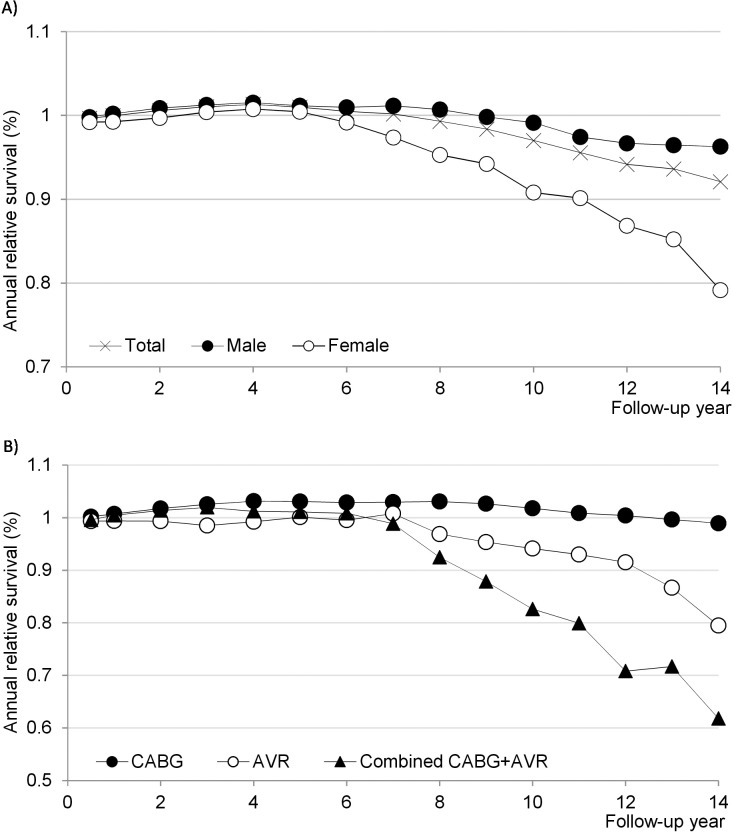
Annual relative survival amongst cardiac surgery patients surviving the first 30 postoperative days. A) Shown in total (n = 8,380) and separately for males (n = 6,244) and females (n = 2,136). B) Shown for patients undergoing isolated coronary artery bypass grafting (CABG, n = 5,593), isolated aortic valve replacement (AVR, n = 699) and combined CABG and AVR (n = 809). A relative survival >1 indicates a survival advantage in the study cohort.

When adjusting for the expected survival in a similar subset of the general Norwegian population, the 1-, 5- and 10-year relative survival rates were estimated to 97.8%, 98.8% and 94.9%, respectively. However, when excluding patients who died within 30 days postoperatively (n = 184), there was a survival benefit in cardiac surgery patients compared to the reference population: Observed survival during the first four years of follow-up was higher than expected survival (relative survival >1, Fig 3A). Survival during the three subsequent years was comparable to that of the background population (relative survival = 1). Overall relative survival decreased from the eighth year and onwards; however, the reduction in survival started earlier and was greater amongst females.

Isolated CABG, AVR and combined AVR and CABG showed similar relative survival from the first throughout the seventh year of follow-up (Fig 3B). However, from the eighth year of follow up, survival was most reduced for combined AVR and CABG, moderately reduced for isolated AVR, whereas relative survival remained >1 for isolated CABG. After the tenth year of follow up, the numbers at risk were too small for statistical analysis.

#### Relative mortality

When comparing the overall observed and expected number of deaths in patients who were still alive after 30 postoperative days, we found that patients undergoing cardiac surgery from 2000 through 2014 did not have significantly different mortality compared to the general population (overall SMR 1.02, 95% CI 0.97–1.06, Table 1). However, subgroup analyses showed that females (SMR 1.17, 95% CI 1.07–1.27) and patients aged <70 years (SMR 1.77, 95% CI 1.52–2.04 for <60 years and SMR 1.17, 95% CI 1.06–1.29 for 60–69 years) had a significantly higher relative mortality when adjusting for background mortality. For men, patients aged >70 years showed a survival benefit. There was a trend that females aged ≥ 80 years may have a similar survival advantage, however, the number of cases in this age group was small.

**Table 1:** Standardized mortality ratios stratified on gender and age group (n = 8,380).

Age group	Total					Males				Females			
	Number at risk (n)	Observed deaths (n)	Expected deaths (n)	SMR	95% CI	Observed deaths (n)	Expected deaths (n)	SMR	95% CI	Observed deaths (n)	Expected deaths (n)	SMR	95% CI
< 60 years	2,081 (24.8%)	179	101	1.77	(1.52–2.04)	152	91	1.67	(1.42–1.96)	27	10	2.61	(1.79–3.81)
60–69 years	2,636 (31.5%)	394	337	1.17	(1.06–1.29)	314	287	1.09	(0.98–1.22)	80	50	1.61	(1.29–2.00)
70–79 years	2,999 (35.8%)	995	998	1.00	(0.94–1.06)	676	752	0.90	(0.83–0.97)	319	246	1.30	(1.16–1.45)
≥ 80 years	664 (7.9%)	292	392	0.74	(0.66–0.84)	175	234	0.75	(0.65–0.87)	117	158	0.74	(0.62–0.89)
Total	8,380	1,860	1,828	1.02	(0.97–1.06)	1,317	1,364	0.97	(0.91–1.02)	543	464	1.17	(1.07–1.27)

CI; confidence interval, SMR; standardized mortality ratio.

Furthermore, stratification by surgical procedure showed that patients undergoing AVR, both isolated and with concomitant CABG, had a higher relative mortality compared to isolated CABG (p<0.001 for both). The findings remained comparable when adjusting for intergroup differences in age and sex distributions. Concomitant CABG in AVR-patients was not associated with a higher relative mortality (p = 0.24).

#### Risk factor analysis

The multivariate Cox PH model showed that observed mortality increased with age (HR per 5 years 1.46 (1.41–1.50), p<0.001). Gender was not an independent predictor of observed long-term mortality following cardiac surgery (p = 0.09). However, following propensity score matching, resulting in standardized differences ≤ 9% for all covariates (S2 Fig), gender emerged as a predictor of observed mortality (HR = 0.81, 95% CI (0.70–0.93), p = 0.004) in the matched 1,493 pairs of females and males (n = 2,986). This association was not reproduced when evaluating cardiovascular mortality (p = 0.43).

Pre-existing chronic heart failure, chronic pulmonary disease, preoperative serum creatinine concentrations >140 μmol/L, peripheral vascular disease, diabetes and current tobacco smoking were associated with increased risk of long-term mortality (Table 2). Mortality increased linearly with increased complexity and number of procedures performed. There were non-proportional hazards over time, as shown by fitting piecewise hazard ratios (Table 2). The type of surgical procedure, co-existing chronic pulmonary disease and reduced kidney function seemed to play more dominant roles during the first year. The predictor estimates remained comparable when selecting patients included until 2006 or 2010. Furthermore, there were only small changes when using cardiovascular death as the outcome variable (S3 Table).

**Table 2:** Risk factors associated with observed mortality.

Predictor	Time period							
	Complete follow-up		≤ 1 year		1–5 years		> 5 years	
	HR	(95% CI)	HR	(95% CI)	HR	(95% CI)	HR	(95% CI)
Age per 5 years	1.46	(1.41–1.50)***	1.25	(1.14–1.36)***	1.37	(1.30–1.44)***	1.53	(1.46–1.60)***
Female gender	0.91	(0.82–1.02)	1.07	(0.77–1.48)	0.68	(0.56–0.83)***	1.03	(0.88–1.20)
Surgical category:								
1) Isolated CABG (reference)	1.00	—	1.00	—	1.00	—	1.00	—
2) 1 non-CABG procedure	1.49	(1.29–1.73)***	2.41	(1.59–3.64)***	1.56	(1.20–2.03)***	1.33	(1.04–1.68)*
3) 2 surgical procedures	1.53	(1.37–1.71)***	2.06	(1.44–2.93)***	1.60	(1.32–1.94)***	1.44	(1.21–1.71)***
4) ≥ 3 surgical procedures	1.94	(1.51–2.48)***	2.43	(1.20–4.90)*	2.17	(1.47–3.20)***	1.53	(1.00–2.33)*
Chronic cardiac insufficiency	1.61	(1.44–1.79)***	1.89	(1.38–2.60)***	1.78	(1.48–2.15)***	1.49	(1.26–1.77)***
Chronic pulmonary disease	1.70	(1.52–1.89)***	2.38	(1.75–3.25)***	1.69	(1.40–2.04)***	1.52	(1.27–1.81)***
Serum creatinine >140 μmol/L	2.07	(1.77–2.42)***	3.02	(2.02–4.53)***	2.00	(1.54–2.60)***	1.85	(1.43–2.41)***
Diabetes mellitus	1.58	(1.40–1.78)***	1.63	(1.15–2.32)**	1.50	(1.23–1.84)***	1.66	(1.38–1.99)***
Peripheral vascular disease	1.69	(1.50–1.91)***	1.16	(0.77–1.73)	1.95	(1.60–2.37)***	1.65	(1.37–2.00)***
Current smoking	1.42	(1.29–1.57)***	1.54	(1.12–2.11)**	1.25	(1.05–1.48)*	1.41	(1.21–1.64)***

Hazard ratios are given for the complete follow-up period, as well as piecewise for the 1^st^ year (n = 8,380), 1^st^-5^th^ year (n = 7,704) and >5^th^ year (n = 5,207) of follow-up. HR; hazard ratio, CI; confidence interval.

*p<0.05

**p<0.01

***p<0.001. CABG; coronary artery bypass grafting.

**Fig 4:**
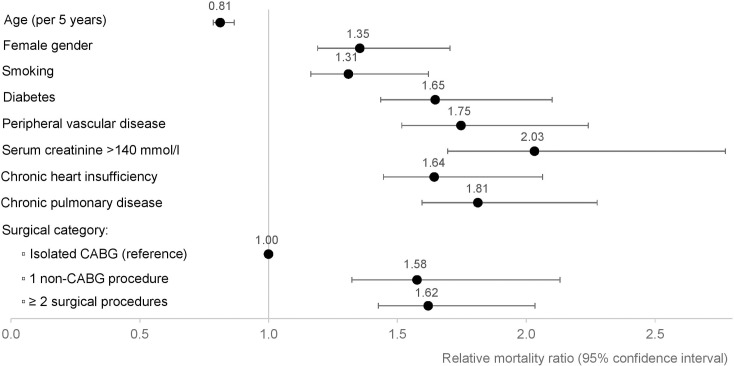
Predictors of relative mortality. Estimated relative mortality ratios (RMR) with corresponding 95% confidence intervals (CI) for predictor variables of long-term relative mortality in patients undergoing cardiac surgery. A ratio of 1 indicates no difference. CABG, coronary artery bypass grafting.

When adjusting for background mortality, relative mortality was higher in younger patients (RMR per 5 years 0.81 (0.79–0.84), p<0.001) and increased with female gender (RMR 1.35 (1.19–1.54), p<0.001). The multivariate analysis showed that the predictors of observed mortality mentioned above were also associated with relative mortality (Fig 4). Results were consistent also for the subgroup analysis including only CABG and AVR patients (S3 Fig).

## Discussion

In the present study cohort, short- and long-term observed mortality rates remained constant throughout the study period. Comparing observed mortality to that expected in a matched sample from the general population, patients undergoing cardiac surgery showed excellent survival throughout the first seven years of follow-up. Subsequently, there was a modest reduction in overall annual survival, which was more pronounced in female patients as well as patients undergoing other procedures than isolated CABG. Confirming these findings, the ratio of observed and expected deaths was higher for females, in younger age groups and in patients undergoing AVR, independently of concomitant CABG. Multivariate survival analyses indicated that the same predictor variables associated with observed mortality remained significant predictors of relative mortality.

### Long-term survival

The good long-term survival in cardiac surgery patients underscores a continued patient benefit from this intervention in appropriately selected patients. Despite an increasing trend of more comorbid and complex patients being referred to cardiac surgery, long-term survival remained unchanged throughout the study period. Furthermore, patients undergoing cardiac surgery in Trondheim also showed similar and even somewhat improved observed and relative survival rates compared to older reports from other centres [15, 16].

Older reports on relative long-term survival were performed on patients undergoing CABG and valvular surgery between the 1970s and 1990s. However, the comparison of present survival rates with older reports have not taken into account the patients’ changing risk profiles. Reasons for the sustained or even improved long-term survival therefore remain uncertain, but might be related to different risk profiles, geographically or temporally, advances in secondary prevention or improvements in medical care.

### Age

In accordance with previous studies, higher age was as an independent predictor of observed long-term mortality, and relative survival analyses revealed that the effect of age was attributable to the population risk [13, 14, 16]. On the contrary, relative mortality increased with decreasing age, also after adjustment for smoking and comorbidities. The need for cardiac surgery at a young age may indicate severe cardiovascular disease, which is progressive and conveys an increased risk for future cardiovascular events.

In general, older patients have reduced functional reserves as well as more systemic and cardiovascular comorbidities; thus having a higher operative risk [39, 40]. However, if they survive the early postoperative phase, our observations support that they show excellent long-term results [40]. Our study indicates that co-existing comorbidities remain the major determinants of surgical eligibility, not chronological age as such; underlining the importance of detailed operative risk stratification in older patients.

### Gender

The role of gender as a risk factor for long-term mortality is debated [41]. In the present multivariate analysis, gender did not significantly affect observed long-term mortality, despite significant differences in the preoperative risk profiles between genders. Our initial findings comply with previous observations where adjustment for potential predictors in a multivariate analysis eliminated the gender differences seen in observed mortality rates [42]. However, results from the propensity score matched analysis suggest that a gender difference may have been masked by residual confounding. In general, female patients showed a worse risk factor profile. Nevertheless, in the matched pairs of females and males, females had a reduced observed mortality. Importantly, our data suggest that this association may be confounded by the better life expectancy of women, because female patients had a significantly higher mortality risk relative to their expected level of survival. Thus, the female survival advantage observed in the general population was obliterated. This phenomenon was also found in an old study from Norway [18], and might be explained by more aggressive heart disease in females [43].

### Surgical procedure

Patients undergoing procedures other than isolated CABG showed higher long-term observed and relative mortality. However, a temporal assessment of relative survival across follow-up time showed that up to the seventh year, cumulative survival was comparable in all patient groups. CABG-patients showed a sustained survival benefit throughout follow-up compared to the general population, whereas there was a trend of reduced relative survival in patients undergoing AVR from the eighth year. Overall relative mortality was significantly higher in patients undergoing isolated AVR or combined AVR and CABG, also after adjustment for different distributions of age, gender and comorbidities. Reduced relative survival in AVR-patients was comparable to an old report from Sweden [13]. Here, the mortality risk increased markedly from the fourth year of follow-up.

There was no significant difference in mortality between patients undergoing AVR and combined AVR and CABG. Due to the low number of patients in these two groups, further investigation is warranted. However, our findings comply with other reports, where adjustment for age, gender and other risk factors eliminated concomitant CABG in AVR-patients as an independent predictor of mortality [13, 44, 45]. It has previously been described a trend of reduced relative survival in combined AVR-CABG patients after 8–10 years compared to isolated AVR [13], but as in our study, this difference did not reach statistical significance.

Compared to isolated CABG, the reduced survival in patients undergoing AVR may be causal (i.e. implying a more aggressive disease), act as a marker for a high-risk patient profile, or be related to follow-up care and suboptimal secondary prevention. Further investigation of this patient group might be important for improving outcomes following cardiac surgery.

With the present covariates, the prognostic difference amongst CABG- and AVR-patients persisted despite adjusting for different preoperative risk profiles. Postoperatively, all patients undergoing CABG are routinely started on lipid-lowering treatment and antiplatelet therapy, with additional guidelines for optimizing treatment of diabetes, hypertension and heart failure. The hazards of hyperlipidaemia [46] and benefits of lipid-lowering treatment [47] have also been demonstrated in AVR-patients, but not yet routinely implemented in clinical practice. Trials of antiplatelet therapy and antihypertensive treatment has also been called for [48]. Improving secondary prevention strategies might therefore represent an opportunity to further improve the quality of care and hence long-term outcomes after AVR.

Altogether, the temporal survival trends and multivariate predictor analysis highlight the prognostic importance of systemic and cardiovascular risk factors above surgical factors. Despite more complex preoperative risk profiles, long-term mortality remained unchanged. This might comply with the gradual reduction in cardiovascular mortality in the Norwegian population; from 41% in 2000 to 29% in 2014 [49]. Nevertheless, it remains the number one cause of death in the population. This supports our finding that the predictors of observed mortality and relative mortality were the same. Patients undergoing cardiac surgery suffer severe and progressive heart disease with a continuous risk of symptomatic events and mortality. The beneficial effects of operation will decline over time, thus risk factor control remains the cornerstone for improving the long-term prognosis of these patients.

### Study limitations

This is a single-centre study based on data from cardiac surgery patients in Trondheim, Norway, as well as expected survival rates from the Norwegian population. Thus, the results may not apply to other institutions and countries. Furthermore, patients undergoing cardiac surgery may have been included in the expected survival rates. As the prevalence of cardiac surgery in the general population is low, this will have had little impact on our estimates.

Despite a total follow-up period of up to 14 years, the median follow-up was 6.4 years. Sensitivity analyses for data between 2000–2006 and 2000–2010 did not alter the main results, indicating that our original findings are robust with respect to inclusion period. Furthermore, neither survival trends nor predictors of long-term mortality changed when using cardiovascular death as an alternate endpoint to all-cause mortality. However, due to the risk of classification errors in cause-of-death records [50, 51], relative survival analyses, permitting comparison with data from the general population, were applied in order to adjust for mortality of other causes in the cardiac surgery patients.

The causal mechanisms underlying increased mortality in younger patients, females and patients undergoing AVR cannot be deduced from the present study. Despite adjustment for varying risk profiles, we cannot exclude that associated predictors may act as markers for other causal relationships and risk factors which were not accounted for in our analysis. The gender differences in preoperative risk profiles and results from repeated calculations in the propensity score matched subset of patients (n = 2,896) may indicate that there was residual confounding. On the other hand, propensity score matching results in smaller groups for comparison due to difficulties in matching of patients with the most extreme scores. Furthermore, the paradoxical combination of more preoperative risk factors in females but reduced observed mortality may indicate that the association was confounded by the longer life expectancy in females, as indicated by our relative survival analyses. The improved relative survival in older patient groups might be due to a chance effect in small groups when the numbers at risk were reduced towards the end of follow-up.

Cardiac surgery patients constitute a heterogeneous group. In order to reduce patient heterogeneity, patients undergoing off-pump surgery have not been enrolled into CaSOS. Consequently, mortality data from the Norwegian Cause of Death Registry were not available for these patients. A sensitivity analysis only including patients undergoing CABG and/or AVR showed consistent results with the complete study cohort. Nevertheless, patients undergoing AVR still constitute a heterogeneous population ranging from young patients with bicuspid valve disease, to elderly patients with degenerative disease complicated by coexisting comorbidities. The low number of patients in these subgroups limited the pairwise comparison and depth of the subgroup analysis. Our main aim was therefore to identify common effects in a large patient group, where the majority suffers cardiovascular disease with many common risk factors.

The present study did not provide any direct information on the survival of surgical patients in comparison with similar patients who did not undergo cardiac surgery. We did not have data on other long-term outcomes, such as complications like bleeding and thromboembolism or quality of life.

## Conclusions

Despite more complex preoperative risk profiles, the observed 30-day, 1-, 3- and 5-year mortality rates in patients undergoing cardiac surgery in Trondheim, Norway, have remained constant both throughout the study period and compared to older reports. Overall, cardiac surgery patients showed comparable survival to that expected in the general Norwegian population, underlining the benefits of cardiac surgery in appropriately selected patients. However, the beneficial effect was more short-lived and relative mortality increased with lower age, in female patients, and patients undergoing AVR or other procedures than isolated CABG. The present findings therefore identify three patient groups that should receive increased attention in order to further improve patient outcomes. A key to improving patient outcomes might lie in closer attention to the underlying, chronic disease and implementation of appropriate preventive strategies.

## Supporting Information

S1 Fig Long-term observed survival. Unadjusted Kaplan-Meier survival curves stratified on the surgical procedure as classified by EuroSCORE II. Due to the low number of patients, the two latter surgical groups were combined (2 and ≥ 3 surgical procedures). The number at risk (n) at the start of even follow-up years are provided.
(TIF)

S2 Fig Standardized difference plot. Absolute standardized differences in covariate means between female and male cardiac surgery patients before and after propensity score matching on preoperative covariates.
(TIF)

S3 Fig Predictors of long-term relative mortality. Comparison of predictor estimates when modelling long-term relative mortality in patients undergoing isolated CABG, isolated AVR or combined AVR and CABG (n = 7,203, hollow circles), with the complete patient samples stratified on EuroSCORE II’s weighted procedures (n = 8,564, black circles). Patients who died within 30 days following surgery have been excluded. *For EuroSCORE’s categories, the two latter surgical groups were combined (2 and ≥3 surgical procedures) due to small patient groups.
(TIF)

S1 Table Comparison of patient characteristics between genders. (DOCX)

S2 Table Comparison of patient characteristics across time. (DOCX)

S3 Table Risk factors associated with observed cardiovascular mortality. (DOCX)
