# Supplementary figures and images for: Reduced Long-Term Relative Survival in Females and Younger Adults Undergoing Cardiac Surgery: A Prospective Cohort Study
Source: PLoS One. 2016 Sep 28;11(9):e0163754. doi: 10.1371/journal.pone.0163754 (PMC5040400; doi:10.1371/journal.pone.0163754)

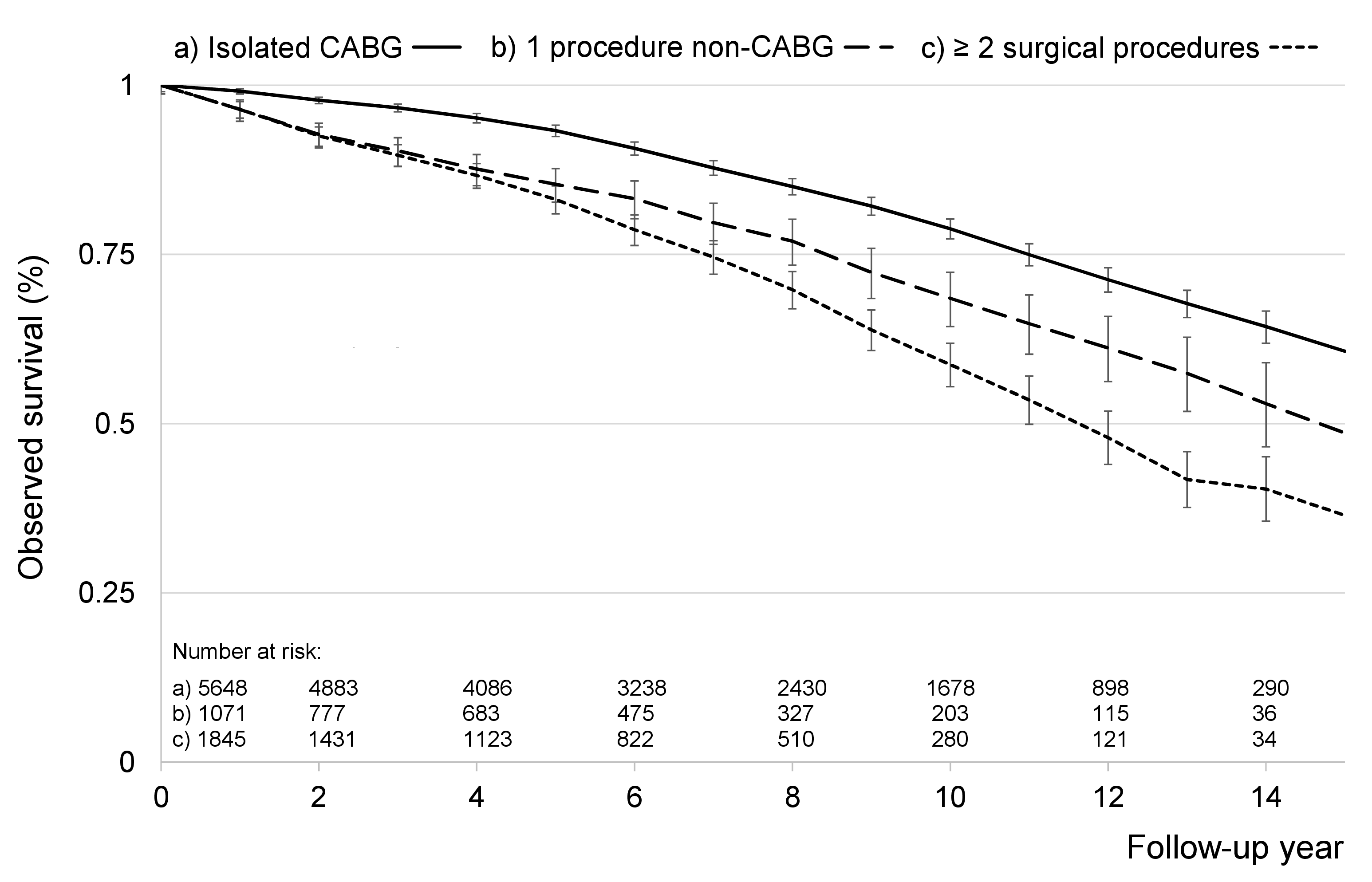

Supplement: S1 Fig — Unadjusted Kaplan-Meier survival curves stratified on the surgical procedure as classified by EuroSCORE II. Due to the low number of patients, the two latter surgical groups were combined (2 and ≥ 3 surgical procedures). The number at risk (n) at the start of even follow-up years are provided. (TIF) [file pone.0163754.s001.tif]

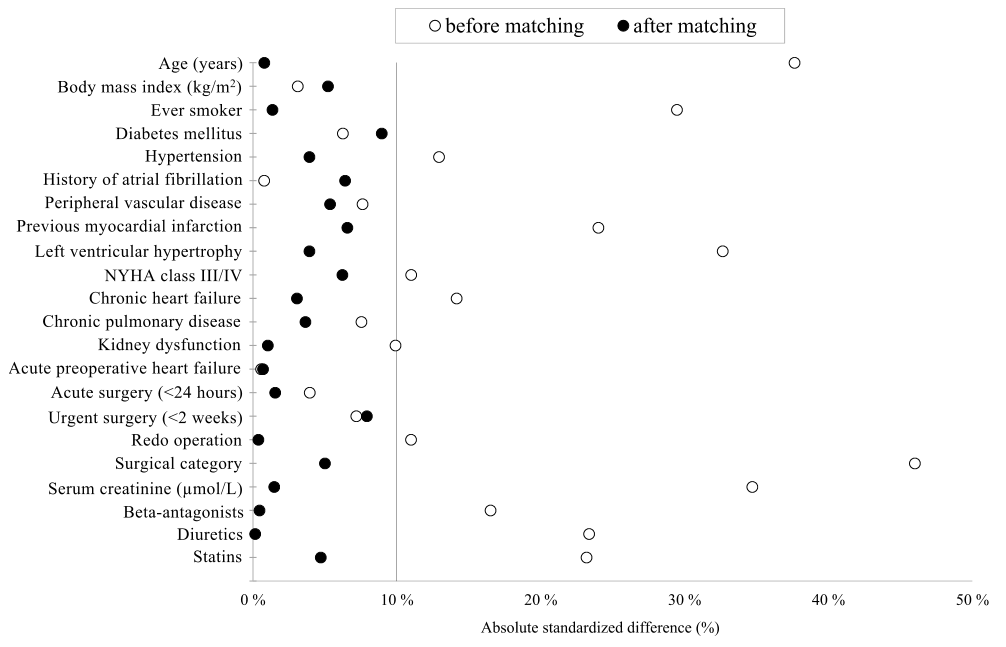

Supplement: S2 Fig — Absolute standardized differences in covariate means between female and male cardiac surgery patients before and after propensity score matching on preoperative covariates. (TIF) [file pone.0163754.s002.tif]

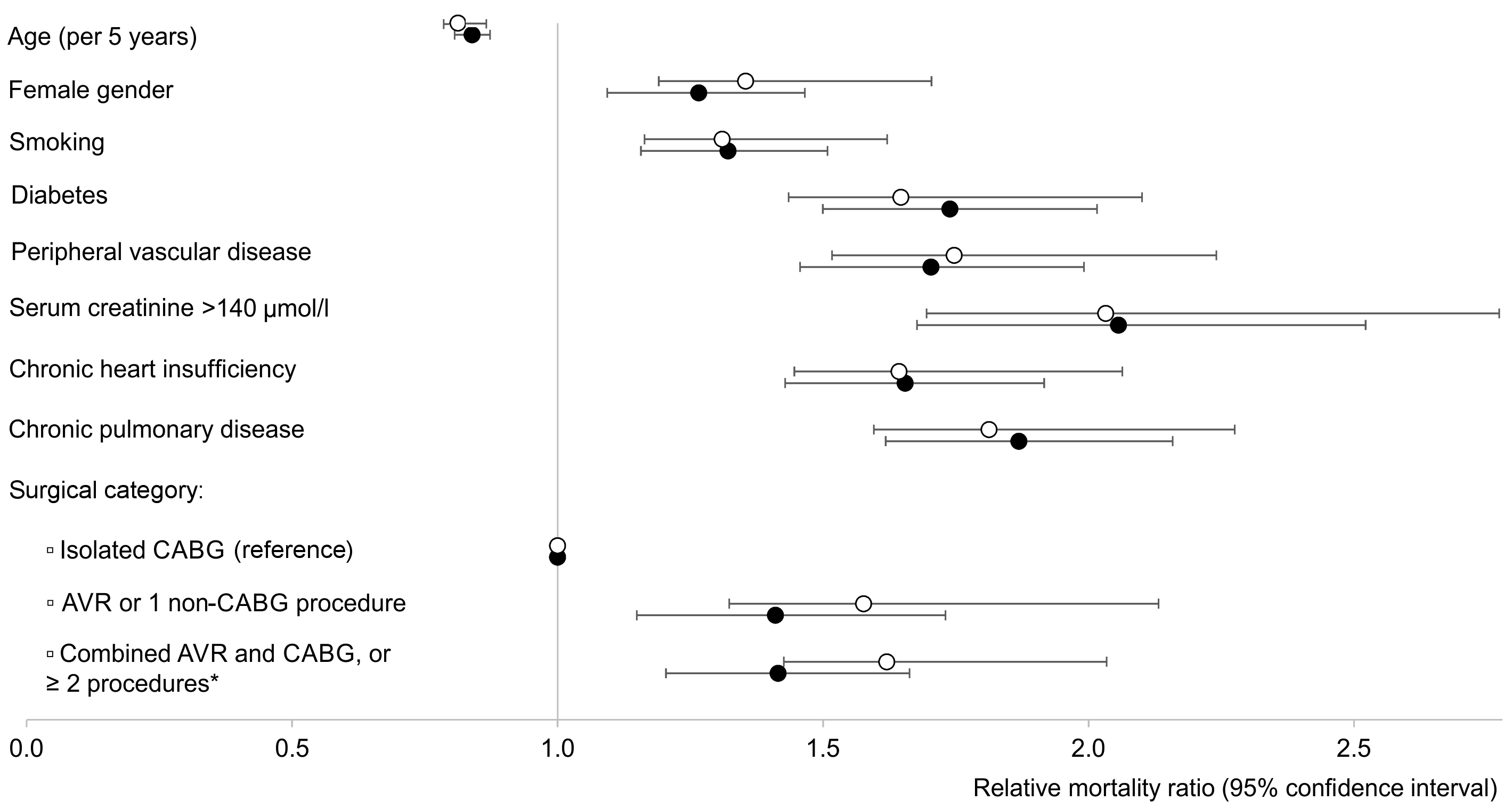

Supplement: S3 Fig — Comparison of predictor estimates when modelling long-term relative mortality in patients undergoing isolated CABG, isolated AVR or combined AVR and CABG (n = 7,203, hollow circles), with the complete patient samples stratified on EuroSCORE II’s weighted procedures (n = 8,564, black circles). Patients who died within 30 days following surgery have been excluded. *For EuroSCORE’s categories, the two latter surgical groups were combined (2 and ≥3 surgical procedures) due to small patient groups. (TIF) [file pone.0163754.s003.tif]
