# Supplementary material for: Reduced Long-Term Relative Survival in Females and Younger Adults Undergoing Cardiac Surgery: A Prospective Cohort Study
Source: PLoS One. 2016 Sep 28;11(9):e0163754. doi: 10.1371/journal.pone.0163754 (PMC5040400; doi:10.1371/journal.pone.0163754)
Supplement: S1 Table — (DOCX) [file pone.0163754.s004.docx]

**S1 Table. Comparison of patient characteristics between genders.**

| **Characteristic** | **Female**  **(n=2,211)** | **Male**  **(n=6,353)** | **P-value** |
| --- | --- | --- | --- |
| Age (years) | 71 (70-71) | 66 (66-66) | <0.001 |
| Body mass index (kg/m^2^) | 26.4 (26.2-26.6) | 26.7 (26.6-26.8) | 0.001 |
| Ever smoker | 961 (48.2%) | 3,656 (63.6%) | <0.001 |
| Diabetes mellitus | 343 (15.5%) | 847 (13.3%) | 0.01 |
| Hypertension | 1,290 (58.3%) | 3,307 (52.1%) | <0.001 |
| History of atrial fibrillation | 285 (12.9%) | 807 (12.7%) | 0.83 |
| Peripheral vascular disease | 197 (8.9%) | 715 (11.3%) | 0.002 |
| Previous myocardial infarction | 810 (36.6%) | 3,058 (48.1%) | <0.001 |
| Left ventricular hypertrophy | 781 (39.4%) | 1,343 (24.2%) | <0.001 |
| NYHA class III/IV | 1,573 (71.2%) | 4,189 (66.0%) | <0.001 |
| Chronic heart failure | 472 (22.5%) | 899 (16.6%) | <0.001 |
| Chronic pulmonary disease | 387 (17.5%) | 933 (14.7%) | 0.002 |
| Kidney dysfunction | 69 (3.1%) | 317 (5.0%) | <0.001 |
| Acute preoperative heart failure | 22 (1.2%) | 63 (1.2%) | 0.99 |
| Acute surgery (<24 hours) | 139 (6.3%) | 318 (5.0%) | 0.02 |
| Urgent surgery (<2 weeks) | 853 (38.6%) | 2,682 (42.2%) | 0.003 |
| Redo operation | 55 (2.5%) | 282 (4.4%) | <0.001 |
| Surgical category |  |  | <0.001 |
| 1. Isolated CABG | 1,136 (51.4%) | 4,512 (71.0%) |  |
| 1. 1 procedure non-CABG | 478 (21.6%) | 593 (9.3%) |  |
| 1. 2 surgical procedures | 529 (23.9%) | 1,088 (17.1%) |  |
| 1. ≥ 3 surgical procedures | 68 (3.1%) | 160 (2.5%) |  |
| Serum creatinine (µmol/L) | 76 (76-77) | 90 (90-91) | <0.001 |
| Beta-blockers** | 1.525 (69.0%) | 4,854 (76.4%) | <0.001 |
| Diuretics** | 778 (35.2%) | 1,567 (24.7%) | <0.001 |
| Statins** | 1,489 (67.4%) | 4,942 (77.8%) | <0.001 |
| Cardiopulmonary bypass time (min) | 82 (80-83) | 78 (77-79) | 0.003 |

Categorical variables are given in n (%), continuous variables in median (95% confidence interval). Differences between genders were tested with χ^2^ test and Mann-Whitney U-test for categorical and continuous data, respectively. **Medication before referral for surgery. CABG; coronary artery bypass grafting, NYHA; New York Heart Association Functional Classification (class I-IV).
