# Supplementary material for: Reduced Long-Term Relative Survival in Females and Younger Adults Undergoing Cardiac Surgery: A Prospective Cohort Study
Source: PLoS One. 2016 Sep 28;11(9):e0163754. doi: 10.1371/journal.pone.0163754 (PMC5040400; doi:10.1371/journal.pone.0163754)
Supplement: S2 Table — (DOCX) [file pone.0163754.s005.docx]

**S2 Table. Comparison of patient characteristics across time.**

|  | Time period | | | P-value |
| --- | --- | --- | --- | --- |
|  | 2000-2004 (n=3,122) | 2005-2009  (n=2,875) | 2010-2014  (n=2,567) |  |
| **Preoperative characteristics** |  |  |  |  |
| Age (years) | 67 (66.5-67.0) | 67 (67.0-67.5) | 67 (66.5-67.0) | 0.22 |
| Female gender | 796 (25.5%) | 774 (26.9%) | 641 (24.9%) | 0.23 |
| Body mass index (kg/m^2^) | 26.3 (26.2-26.4) | 26.8 (26.7-26.9) | 26.8 (26.6-27.0) | <0.001 |
| Ever smoker | 1,686 (54.0%) | 1,525 (53.0%) | 1,406 (54.8%) | 0.44 |
| Diabetes mellitus | 371 (11.9%) | 426 (14.8%) | 393 (15.3%) | <0.001 |
| History of atrial fibrillation | 1,517 (48.6%) | 1,314 (45.7%) | 1,084 (42.2%) | <0.001 |
| Peripheral vascular disease | 353 (11.3%) | 289 (10.1%) | 270 (10.5%) | 0.28 |
| Previous myocardial infarction | 1,437 (46.0%) | 1,298 (45.2%) | 1,132 (44.1%) | 0.35 |
| Chronic pulmonary disease | 443 (14.2%) | 384 (13.4%) | 493 (19.2%) | <0.001 |
| Chronic heart failure | 505 (16.2%) | 402 (14.0%) | 419 (16.3%) | 0.024 |
| Kidney dysfunction | 172 (5.5%) | 111 (3.9%) | 103 (4.0%) | 0.003 |
| Acute preoperative heart failure | 19 (0.6%) | 26 (0.9%) | 40 (1.6%) | 0.001 |
| Acute surgery (<24 hours) | 161 (5.2%) | 155 (5.4%) | 141 (5.5%) | 0.84 |
| Urgent surgery (<2 weeks) | 1,240 (39.7%) | 1,183 (41.2%) | 1,112 (43.3%) | 0.02 |
| Redo operation | 155 (5.0%) | 119 (4.1%) | 63 (2.5%) | <0.001 |
| Surgical category: |  |  |  | <0.001 |
| 1. Isolated CABG | 2,240 (71.2%) | 1,824 (63.4%) | 1,584 (61.7%) |  |
| 1. 1 non-CABG procedure | 321 (10.3%) | 348 (12.1%) | 402 (15.7%) |  |
| 1. 2 surgical procedures | 493 (15.8%) | 602 (20.9%) | 522 (20.3%) |  |
| 1. ≥ 3 surgical procedures | 68 (2.2%) | 101 (3.5%) | 59 (2.3%) |  |
| Serum creatinine (µmol/l) | 95 (95-96) | 82 (81-82) | 81 (80-82) | <0.001 |
| Creatinine clearance* (ml/min) | 73.2 (72.3-74.1) | 87.3 (86.1-88.5) | 89.8 (88.5-91.0) | <0.001 |
| Hemoglobin (g/dl) | 13.8 (13.7-13.8) | 13.8 (13.8-13.9) | 14.0 (13.9-14.0) | <0.001 |
| **Preoperative medications** |  |  |  |  |
| Antiarrythmics | 42 (1.4%) | 70 (2.4%) | 62 (2.4%) | 0.003 |
| Beta-blockers | 2,512 (80.5%) | 2,168 (75.5%) | 1,699 (66.2%) | <0.001 |
| Diuretics | 766 (24.6%) | 769 (26.8%) | 810 (31.6%) | <0.001 |
| Statins | 2,189 (70.1%) | 2,264 (78.9%) | 1,978 (77.1%) | <0.001 |
| **Intraoperative characteristics** |  |  |  |  |
| Cardiopulmonary bypass time (min) | 72 (71-73) | 79 (78-81) | 85 (84-87) | <0.001 |
| Intraoperative red cell transfusion (no/yes) | 433 (13.9%) | 539 (18.8%) | 613 (23.9%) | <0.001 |
| Use of inotropic support (no/yes) | 759 (24.3%) | 676 (23.5%) | 776 (30.2%) | <0.001 |
| Use of vasoconstrictors (no/yes) | 2,115 (67.8%) | 2,667 (92.8%) | 2,501 (97.4%) | <0.001 |
| **Postoperative factors** |  |  |  |  |
| Postoperative hospital stay (days) | 6.5 (6.5-6.5) | 6 (6-6) | 5.5 (5-5.5) | <0.001 |
| Pneumothorax | 92 (3.0%) | 90 (3.1%) | 104 (4.1%) | 0.05 |
| Myocardial infarction | 203 (6.5%) | 160 (5.6%) | 146 (5.9%) | 0.25 |
| Acute kidney injury | 386 (12.4%) | 321 (11.2%) | 297 (11.6%) | 0.35 |
| Sepsis | 26 (0.8%) | 19 (0.7%) | 11 (0.4%) | 0.17 |
| Multi-organ failure | 58 (1.9%) | 61 (2.1%) | 59 (2.3%) | 0.50 |
| 30-day mortality | 73 (2.3%)) | 52 (1.8%) | 59 (2.3%)) | 0.30 |

Categorical variables are given in n (%), continuous variables in median (95% confidence interval). Differences across the study period were tested with χ^2^ and Kruskal-Wallis tests for categorical and continuous data, respectively. *Creatinine clearance calculations based on formula from Cockcroft and Gault [1]. CABG; coronary artery bypass grafting.

**References:**

[1] Cockcroft DW, Gault MH. *Prediction of creatinine clearance from serum creatinine*. Nephron 1976;**16**:31-41.
