# Supplementary material for: Reduced Long-Term Relative Survival in Females and Younger Adults Undergoing Cardiac Surgery: A Prospective Cohort Study
Source: PLoS One. 2016 Sep 28;11(9):e0163754. doi: 10.1371/journal.pone.0163754 (PMC5040400; doi:10.1371/journal.pone.0163754)
Supplement: S3 Table — (DOCX) [file pone.0163754.s006.docx]

S5 Table: Risk factors associated with observed cardiovascular mortality^†^

| **Predictor** | **Time period** | | | | | | | | | | |
| --- | --- | --- | --- | --- | --- | --- | --- | --- | --- | --- | --- |
|  | **Complete follow-up** | |  | **≤ 1 year** | |  | **1-5 years** | |  | **> 5 years** | |
|  | HR | (95% CI) |  | HR | (95% CI) |  | HR | (95% CI) |  | HR | (95% CI) |
| Age per 5 years | 1.43 | (1.37-1.50)*** |  | 1.27 | (1.14-1.42)*** |  | 1.29 | (1.20-1.39)*** |  | 1.58 | (1.48-1.68)*** |
| Female gender | 0.98 | (0.84-1.14) |  | 1.13 | 0.76-1.69) |  | 0.68 | (0.51-0.92)* |  | 1.12 | (0.92-1.36) |
| Surgical category: |  |  |  |  |  |  |  |  |  |  |  |
| Isolated CABG (reference) | 1.00 | --- |  | 1.00 | --- |  | 1.00 | --- |  | 1.00 | --- |
| 1 non-CABG procedure | 1.59 | (1.28-1.98)*** |  | 2.49 | (1.48-4.19)*** |  | 2.10 | (1.43-3.08)*** |  | 1.20 | (0.88-1.64) |
| 2 surgical procedures | 1.78 | (1.52-2.09)*** |  | 2.34 | (1.51-3.63)*** |  | 2.11 | (1.58-2.82)*** |  | 1.55 | (1-24.1.93)*** |
| ≥ 3 surgical procedures | 2.20 | (1.55-3.11)*** |  | 3.53 | (1.64-7.58)*** |  | 2.60 | (1.46-4.63)*** |  | 1.63 | (0.95-2.79) |
| Chronic cardiac insufficiency | 1.84 | (1.58-2.15)*** |  | 1.94 | (1.31-2.87)*** |  | 2.04 | (1.55-2.68)*** |  | 1.72 | (1.39-2.13)*** |
| Chronic pulmonary disease | 1.46 | (1.24-1.72)*** |  | 2.08 | (1.41-3.06)*** |  | 1.39 | (1.04-1.87)* |  | 1.31 | (1.04-1.66)* |
| Serum creatinine >140 µmol/L | 2.17 | (1.73-2.71)*** |  | 3.02 | (1.83-4.98)*** |  | 1.81 | (1.21-2.72)** |  | 2.18 | (1.59-3.00)*** |
| Diabetes mellitus | 1.56 | (1.31-1.85)*** |  | 1.61 | (1.04-2.50)* |  | 1.56 | (1.16-2.12)** |  | 1.52 | (1.20-1.94)*** |
| Peripheral vascular disease | 1.90 | (1.60-2.25)*** |  | 0.97 | (0.57-1.65) |  | 2.48 | (1.86-3.31)*** |  | 1.89 | (1.49-2.40)*** |
| Current smoking | 1.41 | (1.22-1.63)*** |  | 1.72 | (1.16-2.55)** |  | 1.30 | (1.00-1.69)* |  | 1.42 | (1.17-1.72)*** |
| Complete data (n) | 8356 |  |  | 8356 |  |  | 7682 |  |  | 5188 |  |
| All-cause deaths during interval (n) | 1856 |  |  | 182 |  |  | 601 |  |  | 789 |  |
| Circulatory deaths during interval^†^ (n) | 872 |  |  | 119 |  |  | 261 |  |  | 492 |  |

Hazard ratios are given for the complete follow-up period, as well as piecewise for the 1^st^ year (n=8,380), 1^st^-5^th^ year (n=7,704) and >5^th^ year (n=5,207) of follow-up. CABG; coronary artery bypass grafting, CI; confidence interval. HR; hazard ratio. *p<0.05; **p<0.01; ***p<0.001. ^†^As classified according to the International Classification of Diseases (ICD)-10 (chapter IX, block I00-I99) and registered in the Norwegian Cause of Death Registry.
